# Cardiac Arrest Due to Cyanide Intake

**DOI:** 10.7759/cureus.12894

**Published:** 2021-01-25

**Authors:** Murat Seyit, Atakan Yilmaz, Mert Ozen, Serife Ornek, Orkun Gursoy

**Affiliations:** 1 Emergency Medicine, Pamukkale University, Denizli, TUR; 2 Emergency, Pamukkale University, Denizli, TUR

**Keywords:** cyanide, out-of-hospital cardiac arrest, emergency service

## Abstract

Suicidal attempts are the primary cause of cyanide intake, and a majority of these attempts are fatal. Cyanide-induced cardiac arrest or hypotension is common, though the administration of antidotal therapy in patients is not. The patient drank the cyanide ordered from the internet in an unknown amount 10-15 min before being taken to the ED. He informed his friend after taking it, and he collapsed shortly after his friend notified the Emergency Medical Services (EMS). Acute cyanide poisoning, whose rapid detection is vital but difficult to diagnose, leads to rapid hemodynamic and neurological dysfunction. Bitter almond odor and cherry red skin appearance should be the warning signs in the diagnosis of acute cyanide toxicity.

## Introduction

Suicidal attempts are the primary cause of cyanide intake, and a majority of these attempts are fatal. Cyanide-induced hypotension or cardiac arrest is common, though the administration of antidotal therapy in patients is not [1]. Cyanide-associated cardiac arrest may be reported more frequently than expected, and early empirical antidotal treatment, such as hydroxycobalamin, may improve survival for those exposed to cyanide [2]. Although cyanide is involved in a reaction with more than 40 metalloenzymes, its fatal effect is noncompetitive inhibition of cytochrome c oxidase, which impedes cellular respiration and triggers hypoxic anoxia [3].

## Case presentation

**Figure 1:**
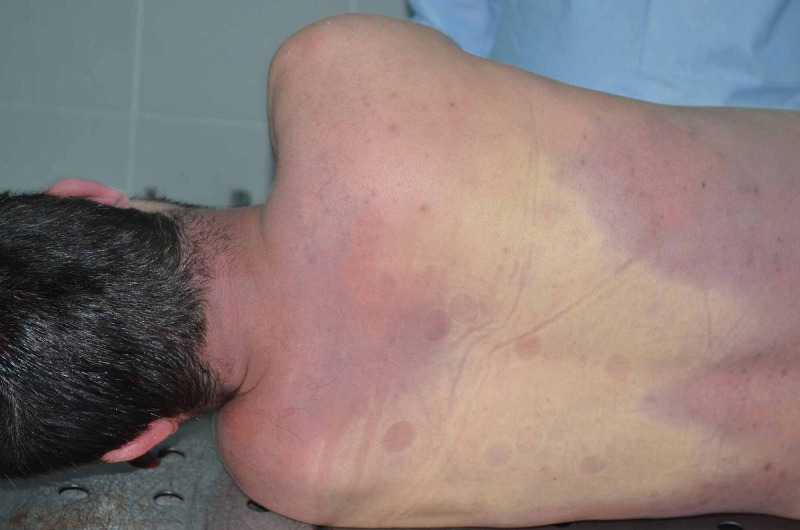
Diffused bright redness was identified on his upper body and back.

A male, 26-year-old patient with a cardio pulmonary arrest was admitted to ED at midnight Emergency Medical Services (EMS). In the light of the information from the patient's relative, it was found out that he drank the cyanide ordered from the internet in an unknown amount. He informed his friend within minutes of taking it. It was also reported that when the EMS team arrived at the scene, the patient had a cardio pulmonary arrest and was brought to the ER with cardio pulmonary resuscitation (CPR) by the EMS team. The patient was intubated upon seeing that Glasgow Coma Scale (GCS) score was E1V1M1 during the examination on arrival. The patient, whose blood pressure and pulse could not be measured, was diagnosed with cyanosis and observed to give off a different odor. When the patient's rhythm was monitored and followed up, he was in the state of asystole, and his neurological examination revealed that his pupils were fixed and dilated. While CPR was being performed on the patient, the National Poison Center, to which the required information about the patient was provided, was contacted for consultation. Upon their suggestion, two IV cannulas were placed, normal saline was administered, and a nasogastric tube was inserted, accompanied by activated charcoal. Beheptal® (Osel Drug Industry and Trade. Inc., Istanbul, Turkey) (cyanocobalamin 30 mcg/2 mL, thiamine 25 mg/2 mL, riboflavin 5 mg/2 mL, pyridoxine 10 mg/2 mL, dexpanthenol 5 mg/2 mL, nicotinamide 50 mg/2 mL) containing cyanocobalamin was administered to the patient. The patient's blood values were calculated as glucose = 261 mg/dL, creatine = 1.6 mg/dL, and sodium = 151 mEq/L. Potassium-alanine aminotransferase (ALT) - aspartate aminotransferase (AST) and blood gas manifested hemolysis. In addition, the ethanol level in the blood turned out to be negative. Once the patient did not respond to approximately one hour of CPR, he was regarded as dead and transferred to the morgue. Furthermore, it is an important diagnostic finding that diffused bright redness was identified on his upper body and back (Figure 1), and his autopsy level of cyanide was 11.59 mg/L (lethal dose > 0.77 mg/L).

## Discussion

Parker-Cote et al. [4] identified a great number of patients in their case reports and case report series on acute cyanide intoxication compiled from different databases. Out of these 102 patients reported in their review study, 86 took cyanide orally. They also found that 78% of the patients were unresponsive, 73% had respiratory failure, 72% had arrhythmia, and 54% were hypotensive. Moreover, 20% of these patients suffered cardiac arrest and 20% epileptic seizures, while 15% had cyanosis, 11% bright red skin, and 15% a noticeable odor. In most cases (66%), the patients needed mechanical ventilation and intubation, while 39% developed refractory hypotension requiring vasopressor support, and 26% were dead. As far as our case is concerned, the patient was brought to the hospital in a state of cardiac arrest, unresponsiveness, and asystole. His blood pressure could not be measured, and he had cyanosis. After death, the patient was reported to give off a bitter almond odor and had bright red areas on his skin.

In a swine model of cyanide-induced cardiac arrest, Bebarta [5] et al. concluded that both IV hydroxocobalamin and epinephrine separately increased survival rate, in comparison to saline solution. Besides, it is suggested that hydroxocobalamin enhanced average blood pressure and pH, reducing blood lactate and cyanide levels as well as the administration of additional epinephrine therapy in comparison to that in the epinephrine group. When the patient in our case was admitted with a cardiac arrest, epinephrine along with Beheptal®, including cyanocobalamin, was administered during CPR, but he did not respond to the therapy.

## Conclusions

Used in a wide range of fields in industry, cyanide is an easy-to-obtain item. Cyanide toxicity may be encountered in several cases, such as leatherwork, pesticide production, photography, metallurgical industry, and exposure to fire. Acute cyanide poisoning, whose rapid detection is vital but difficult to diagnose, leads to rapid hemodynamic and neurological dysfunction. Bitter almond odor and cherry red skin appearance should be the warning signs in the diagnosis of acute cyanide toxicity.
